# Lymphangioleiomyomatosis in patients with tuberous sclerosis: a national centre audit

**DOI:** 10.1186/s13023-024-03115-y

**Published:** 2024-03-26

**Authors:** Jan Johnson, Wendy Somerfield, Simon R. Johnson

**Affiliations:** 1 Centre for Respiratory Research, Translational Medical Sciences and Biodiscovery Institute, School of Medicine, University of Nottingham, Nottingham, UK; 2 National Centre for Lymphangioleiomyomatosis, Nottingham University Hospitals NHS Trust, Nottingham, UK; 3 Nottingham NIHR Biomedical Research Centre, Nottingham, UK

**Keywords:** Tuberous sclerosis complex, Lymphangioleiomyomatosis, Screening, mTOR inhibitor, Pneumothorax, Angiomyolipoma, Epilepsy

## Abstract

**Background:**

Lymphangioleiomyomatosis (LAM) is common in tuberous sclerosis complex (TSC) yet under recognised with management mostly based upon evidence obtained from patients with sporadic LAM. We performed a prospective audit of patients with TSC-LAM attending a national referral centre to inform management guidelines.

**Methods:**

The UK LAM Centre was established in 2011 and conducts a prospective audit of pre-defined quality outcomes for all subjects. Audit data are reported on all patients with TSC-LAM and a comparator population of patients with sporadic LAM.

**Results:**

Between 2011 and 2022, 73 patients were seen with TSC-LAM. All were women with a mean (SD) age of 39 (12) years. Referral rates were similar over the study period including after the introduction of CT screening. Median age of diagnosis with TSC was 11 years (range 0–70) with one third diagnosed with TSC as adults. Compared with all TSC patients in the ‘TOSCA’ registry, TSC-LAM patients tended to have been diagnosed with TSC at an older age, had fewer neuro-cognitive manifestations and were more likely to have angiomyolipoma. The most common presentations of TSC-LAM were following workup for angiomyolipoma, pneumothorax or dyspnoea with only one fifth detected after CT screening. Baseline FEV_1_ and DL_CO_ at first assessment were reduced to 77 and 63% predicted respectively and were similar to patients with sporadic LAM. During follow-up, FEV_1_ fell by a mean of 81 ml/year and DL_CO_ fell by 0.309 mmol/ml/kPa/year in patients not being treated with an mTOR inhibitor. 55% required treatment with either sirolimus or Everolimus for LAM or angiomyolipoma respectively. For those treated with an mTOR inhibitor, mean FEV_1_ fell by 3 ml/year and DL_CO_ increased by 0.032 mmol/ml/kPa/year and was similar to sporadic LAM. Risk of death due to LAM or need for lung transplant in patients with TSC-LAM was 0.67%/year.

**Conclusions:**

Despite screening recommendations, LAM is often diagnosed in TSC after symptoms develop which may delay treatment. Complications including pneumothorax and loss of lung function are significant and similar to sporadic LAM. Work is needed to implement the recommended CT screening for LAM and improve respiratory care for TSC-LAM.

## Introduction

Tuberous sclerosis complex (TSC) is an autosomal dominant genetic disorder which affects around 1 in 6000 live births [1]. The disease is characterised by hamartomas in multiple organ systems, particularly the central nervous system, skin, heart, kidneys and lungs [2]. The varying occurrence of TSC hamartomas can result in differing disease manifestations and severities even within families [3]. Presentation is dependent upon the organ systems affected although the majority of patients present in infancy due to skin features, particularly hypomelanotic patches and angiofibromas or cerebral involvement causing epilepsy, intellectual impairment, autism and attention deficit hyperactivity disorder [4]. Disease features evolve with age, and angiomyolipoma, a benign tumour occurring mostly in the kidneys, tends to occur in adolescents and younger adults eventually affecting around 80% of patients [5]. Lymphangioleiomyomatosis (LAM) is a lung and lymphatic manifestation of TSC with an onset in younger adults, most commonly women which becomes more prevalent with increasing age [4]. LAM results in the accumulation of lung cysts and lymphatic abnormalities, progressively reducing lung function resulting in dyspnoea, pneumothorax, chylous collections and in some cases respiratory failure.

Until relatively recently LAM was thought to occur infrequently in TSC, considered of lesser importance and is still significantly under-diagnosed [6]. However, systematic studies using CT scanning show that lung cysts consistent with LAM are present in half of women with TSC and 10–30% of men, with most women with TSC having some lung cysts by 40 years [7–9]. For a minority of patients, often with milder cerebral manifestations, a diagnosis of TSC is delayed until adulthood after presentation with LAM or angiomyolipoma which may lead to significant morbidity, with recent large tertiary referral series suggesting that LAM is a leading cause of death among adult women with TSC [10, 11].

LAM also occurs as a rare sporadic disease in the absence of TSC where somatic mutations in *TSC2* are restricted to LAM cells [12]. Sporadic LAM affects almost exclusively women with a prevalence of around 9/million women [13]. The lung and lymphatic phenotype of TSC and sporadic LAM are identical and additionally, angiomyolipoma also occur in half of patients with sporadic LAM, suggesting that sporadic LAM may represent mosaicism in TSC [14, 15]. TSC-LAM was previously thought to run a more indolent course than sporadic-LAM although analysis of patients matched for age and disease duration suggest the rates of loss of lung function are similar [16, 17]. Whilst the prevalence of TSC predicts that TSC-LAM should be more common than sporadic LAM, women with sporadic LAM make up the greater proportion of people receiving care at specialised LAM services [18, 19]. Consequently, current management guidelines for LAM tend to be based on studies of patients with sporadic LAM. Since 2013, international TSC guidelines have recommended CT screening for LAM in women with TSC from 18 years [20]. As this is likely to identify more patients with TSC-LAM it is important to understand the clinical phenotype of these patients to both tailor treatment appropriately and ensure that LAM is detected early to minimise lung function loss. We therefore conducted an audit of patients with TSC-LAM referred to a national tertiary centre to understand how the clinical manifestations of TSC-LAM compared with sporadic LAM and how their TSC features compared with a wider group of TSC patients to aid recognition of LAM in TSC.

## Methods

The audit was performed at the National Centre for lymphangioleiomyomatosis, at Nottingham University Hospitals NHS Trust, Nottingham UK. The Centre was established in 2011 as the single national centre to provide comprehensive clinical care for UK patients with LAM including TSC-LAM and is funded by NHS England highly specialised commissioning. When commissioned, pre-specified quality outcomes were defined to monitor centre performance including rate of lung function loss, rates of pneumothorax and angiomyolipoma haemorrhage, mTOR inhibitor use, lung transplant referrals and survival. These outcomes are subject to a prospective continuous audit and reported to NHS England.

All patients with TSC-LAM who were evaluated at the Centre between 2011 and 2022 were included. Patients with both sporadic and TSC-LAM undergo a comprehensive initial assessment as part of routine clinical care including a detailed history incorporating features of TSC, examination of the skin for signs of TSC, CT thorax, and cross-sectional imaging of the abdomen using CT or MRI. Lung function testing (FEV_1_ and DL_CO_) is performed according to current ATS/ERS standards [21] at a single laboratory by experienced technicians. Lung function measurements are not reported in cases where disability prevented reproducible results being obtained. Follow up visits are determined by clinical need at which clinical outcomes are recorded and FEV_1_ and DL_CO_ are repeated.

A comparison group of patients with sporadic LAM evaluated at the UK LAM Centre and cared for in the same way but without TSC was also studied. This group comprised all 175 subjects with definite sporadic LAM evaluated over the same period. All subjects had baseline phenotype and lung function data and 152 had follow-up data. LAM was defined by ATS/JRS guidelines in both groups [22].

Clinical features of LAM and TSC were obtained from medical notes. Subjects were grouped by their main symptom at presentation categorised as one of dyspnoea, pneumothorax, haemoptysis, angiomyolipoma related, screened as part of TSC management, chance (when LAM is discovered during investigation of unrelated symptoms) or any other symptom. Subjects were also grouped according to the individual clinical manifestations of LAM, categorised as the presence of lymphatic involvement (chylous collections, lymphadenopathy or lymphatic masses), angiomyolipoma or pneumothorax. The need for intervention for angiomyolipoma (embolisation or any surgical procedure) and pneumothorax (any surgical intervention) are prespecified LAM Centre quality outcomes and were recorded for each patient.

Data were tested for normality using the Kolmogorov–Smirnov test and differences between means analysed using unpaired t-tests. The presence or absence of phenotypic traits within groups was compared using Fisher’s exact test. Change in lung function for individuals was calculated by the slope of a regression line of all values of FEV_1_ (ΔFEV_1_) or DL_CO_ (ΔDL_CO_) stratified by the use of an mTOR inhibitor [23]. Statistical analyses were performed using Excel (Microsoft corporation), with a *p* value of 0.05 accepted as significant.

## Results

**Fig. 1:**
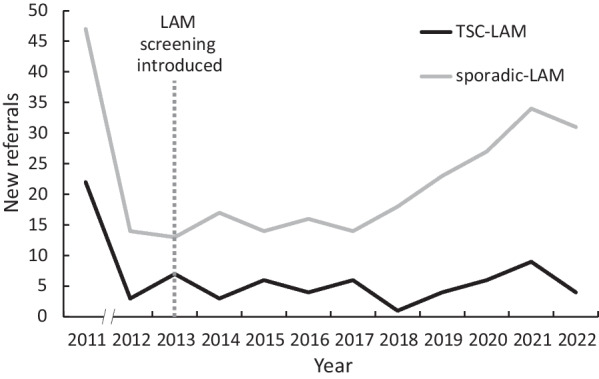
Referral rates of TSC and sporadic LAM cohorts. 2011 includes existing patients transferred to the LAM Centre

Seventy-five patients were seen at the UK LAM Centre for evaluation of TSC-LAM between 2011 and 2022. All were women and had a mean (SD) age of 39 (12) years at first assessment, 73 of these patients had LAM. A control cohort of patients with sporadic LAM seen at the same centre over the same period were used for comparison with the TSC-LAM cohort. The sporadic LAM cohort comprised 175 patients with definite LAM according to ATS/JRS criteria who were participating in the BioLAM observational research cohort. Sporadic LAM patients were all women, with a mean (SD) age of 47 (11) years at first assessment. The rate of referral to the service for patients with TSC-LAM was similar over the study period and did not change after the introduction of CT screening for LAM in 2013 (Fig. 1).

### TSC related characteristics

The mean age of diagnosis with TSC was 17 years (median 11 years, range 0 to 70). One third of these patients were not diagnosed with TSC until adulthood including 12% after 40 years of age. Of these patients, a history of epilepsy was present in 51%, learning difficulty in 24%, TAND in 15% and SEGA in 11%. 95% of the cohort had one or more renal angiomyolipoma and 19% had renal cysts. LAM was present in all but two subjects (97%). Multifocal micronodular pneumocyte hyperplasia (MMPH) was present in 8% and co-existed with LAM in all cases. 19 patients had undergone TSC genotyping with 89% of these patients having a mutation in *TSC2* (Table 1).

**Table 1:** Features of TSC at first LAM Centre assessment in subjects investigated for LAM

Demographic data	value	n
Male/female (%)	0/100	75
Age at 1st assessment (years (SD))	39 (12)	71
Age at TSC diagnosis (years (SD))	17 (18)	62
Genotype (% *TSC1*/*TSC2*/NMI)	10.5/89.5/0	19

TSC feature		% of cohort (n = 75)
Epilepsy		51
Learning difficulty		24
TAND		15
SEGA		11
Angiomyolipoma		95
Renal cysts		19
MMPH		8
LAM		97

Age at LAM diagnosis		% of cohort (n = 71)
< 18 years		1.4
18–40 years		56.3
> 40 years		42.3

n = number of the cohort with this data available. % = percent of cohort with this feature present where data is available.

*TSC* tuberous sclerosis complex. *TSC1 TSC1* mutation detected. *TSC2 TSC2* mutation detected. *NMI* no mutation identified. *TAND* tuberous sclerosis associated neuropsychiatric disorders. *MMPH* multifocal micronodular pneumocyte hyperplasia.

**Fig. 2:**
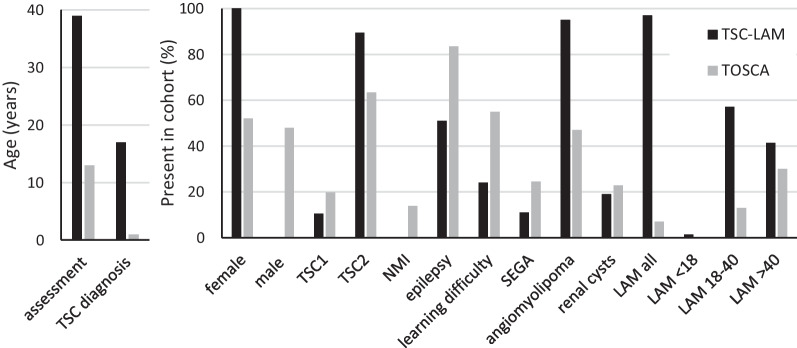
Comparison of clinical features of TSC-LAM cohorts with the TOSCA registry. 73 patients with TSC-LAM are compared with 2093 patients from the TOSCA registry. Median age at assessment and age at diagnosis of TSC in the left panel and percentage of cohort with each disease related feature in the right panel. *TOSCA* the Tuberous Sclerosis registry to increase disease awareness. *TSC* tuberous sclerosis complex. *TSC1* TSC1 mutation detected. *TSC2* TSC2 mutation. *NMI* no mutation identified. *TAND* Tuberous sclerosis associated neuropsychiatric disorders. *MMNPH* Multifocal micronodular pneumocyte hyperplasia

We compared the TSC-LAM patients evaluated at the LAM Centre with 2093 contemporaneous TSC patients currently receiving care for TSC from 31 countries recorded in the *Tuberous Sclerosis registry to increase disease awareness* (TOSCA) [24]. Compared with patients registered in TOSCA, our cohort being evaluated for LAM tended to have been diagnosed with TSC at an older age, had a lower prevalence of neurological and cognitive manifestations, were more likely to have angiomyolipoma and more likely to have *TSC2* mutations than *TSC1* or no identifiable mutation (Fig. 2).

### Presentation of LAM in TSC

We examined the presenting problems leading to a diagnosis of LAM in TSC. Most common was the discovery of LAM due to workup or management of angiomyolipoma which occurred in over a quarter of patients. Pneumothorax and dyspnoea were also common modes of presentation. CT scanning performed to investigate other respiratory and unrelated symptoms also led to the diagnosis in a smaller number of subjects (Table 2). One fifth of patients had been diagnosed after CT screening for LAM. Of all 75 patients with TSC, CT screening for LAM was not performed in 48 and screening status was unknown in a further 10 patients. Seventeen patients were screened for LAM, of these, LAM was present in 13 including four patients diagnosed with TSC as adults, one patient had negative screening at age 33 and developed LAM symptoms at age 41, a further patient had negative screening as a teenager and went on to develop LAM symptoms age 25. Two patients screened did not have LAM.

**Table 2:** Features of LAM at presentation

	TSC-LAM		Sporadic LAM		*p*
	Mean (SD)	n	Mean (SD)	n	
Age at assessment† (years, SD)	39 (12)	71	47 (11)	152	4.06 × 10^−6^
Age first symptom† (years, SD)	31 (11)	62	39 (12)	152	5.27 × 10^−5^

First symptom*	% of cohort (n = 71)	% of cohort (n = 152)	*p*
Dyspnoea	15	31	0.0144
Pneumothorax	23	29	0.336
Haemoptysis	3	3	1
Other respiratory	3	6	0.5092
Angiomyolipoma	28	22	0.3135
Other non-respiratory	4	1	0.0963
Screened	20	0	< 0.00001
Chance	4	7	0.7598

Clinical feature ever present**	% of cohort (n = 73)	% of cohort (n = 152)	*p*
Pneumothorax	52	45	0.393
Pneumothorax surgery	44	22	0.001
Angiomyolipoma	95	61	< 0.00001
Angiomyolipoma treatment	45	14	< 0.00001
Lymphatic	12	20	0.192

Baseline lung function†	Mean (SD)	n	Mean (SD)	n	*p*
FEV_1_ (mean % predicted, SD)	77 (26)	59	73 (25)	152	0.285
DL_CO_ (mean % predicted, SD)	63 (25)	61	57 (18)	151	0.06

Serum VEGF-D†	Mean (SD)	n	Mean (SD)	n	*p*
VEGF-D (pg/ml, mean, SD)	1805 (1508)	28	1585 (3764)	147	0.76

n number of patients with data for this feature.

†mean (standard deviation) analysed by unpaired t-test.

*only one symptom allowed, percent of cohort analysed by Fisher’s exact test. Screened = LAM identified after CT scanning as suggested by TSC guidelines. ** = any number allowed; percent of cohort analysed by Fisher’s exact test.

As most evidence and clinical guidelines for the management of LAM are derived from patients with sporadic LAM [25–27], we compared the clinical phenotype of TSC-LAM with that of sporadic LAM to determine how similar the disease manifestations were. Compared with sporadic LAM, patients with TSC-LAM were younger at their first symptom, less likely to present with dyspnoea, more likely to have angiomyolipoma, and almost twice as likely to require embolization or surgery for angiomyolipoma and surgical treatment for pneumothorax. FEV_1_ and DL_CO_ at first assessment were not significantly different between the two groups (Table 2).

### Outcomes

Patients with TSC-LAM were followed up over a mean duration of 49.2 months and sporadic LAM 82.3 months. Over this period, six (8.2%) patients with TSC-LAM died, in only one case due to LAM and one (1.4%) received a lung transplant. The risk of lung transplantation or death due to LAM in the TSC-LAM patients was 0.67% / year. Patients with TSC had a higher overall mortality than patients with sporadic LAM (*p* < 0.0001). Whilst only one patient in each group died due to LAM, the combined risk of death or transplant per year of observation was statistically higher in patients with TSC-LAM (Table 3).

**Table 3:** LAM and related outcomes

Survival*	TSC-LAM	Sporadic LAM	*p*
	% of cohort (n = 73)	% of cohort (n = 152)	
Alive	92	97	0.081
Died all causes	8.2	2.7	0.083
Died LAM	1.4	0.7	0.548
Lung transplant	1.4	0.7	0.548
Died LAM or lung transplant/year	0.67	0.19	< 0.00001
Died all causes/year	2	0.39	< 0.00001
Follow up duration (months)	49.2	82.3	5.7 × 10^–6^

mTOR inhibitor indication	% of cohort (n = 73)	% of cohort (n = 152)	*p*
All	53	43	0.118
Lung	36	43	0.388
Angiomyolipoma	17	0	< 0.00001

Lung function change	mean (SD)	n	mean (SD)	n	*p*
*no mTOR inhibitor†*					
FEV_1_ (ml/year)	− 81 (398)	29	− 65 (171)	111	0.743
DL_CO_ (mmol/ml/kpa/year)	− 0.309 (1.09)	29	− 0.079 (0.55)	109	0.118
Follow up duration (months)	47.5 (42.2)	28	62.1 (45.4)	111	0.126
Number of measurements	5 (3)	29	6 (4)	111	0.065
*mTOR inhibitor treated†*					
FEV_1_ (ml/year)	− 3 (138)	25	0 (94)	80	0.932
DL_CO_ (mmol/ml/kpa/year)	0.032 (0.32)	23	− 0.081 (0.26)	80	0.082
Follow up duration (months)	47.7 (36.1)	25	65.4 (35.9)	80	0.034
Number of measurements	6 (4)	25	9 (6)	80	0.025

n = number of patients with data for this feature.

*Analysed by Fisher’s exact test.

†Mean (standard deviation) analysed by unpaired t-test.

Fifty-three percent required treatment with an mTOR inhibitor, 36% with sirolimus for LAM and 17% with Everolimus for angiomyolipoma (Table 3). mTOR inhibitors were initiated for TSC-LAM at a mean (SD) age of 39.2 (11) years, a mean of five years earlier than those with sporadic LAM with a mean (SD) age of 44 (9) years (*p* = 0.009).

Serial lung function was measured as part of clinical care. Despite lung function being attempted by experienced lung function technicians, 11 of the 75 patients with TSC were unable to perform lung function reliably and are not reported. For those patients with serial lung function data, FEV_1_ fell by a mean of 81 ml/year and DL_CO_ fell by 0.309 mmol/ml/kPa/year in patients not being treated with an mTOR inhibitor.

Rates of mTOR inhibitor treatment for LAM were similar between the two groups. As some patients with TSC also required mTOR inhibitor treatment for angiomyolipoma, patients with TSC-LAM were more likely to be treated with an mTOR inhibitor overall. The choice of mTOR inhibitor used was largely determined by treatment indication: patients with active lung disease were prescribed sirolimus and patients with renal angiomyolipoma requiring treatment were generally treated with Everolimus. Of the 46 patients with TSC prescribed an mTOR inhibitor, 16 (21%) were prescribed Everolimus, all but one of these for renal angiomyolipoma, with one patient taking the drug for combined renal and lung disease. Thirty (40%) were prescribed sirolimus, 25 of whom were treated for LAM, the remainder for angiomyolipoma and of these 2 had changed from Everolimus to sirolimus (one due to funding issues and one after an adverse reaction). Lung function was lower in patients treated with sirolimus (mean (SD) percent predicted FEV_1_ 59.9 (23), DL_CO_ 48.0 (18)) compared to those treated with Everolimus (FEV_1_ 80.4 (20), DL_CO_ 66.8 (27), both *p* = 0.01). For those treated with either sirolimus or Everolimus, mean FEV_1_ fell by 3 ml/year and DL_CO_ increased by 0.032 mmol/ml/kPa/year. Lung function decline was similar between patients with TSC-LAM and sporadic LAM in both mTOR inhibitor treated and untreated patients (Table 3).

## Discussion

We observed that adult women with TSC suffered significant morbidity from LAM including pneumothorax, lung function decline, and in some cases death from respiratory failure. Despite international TSC guidelines recommending screening of adult women with TSC for LAM [20], over 75% of TSC patients in the current cohort were diagnosed with LAM after developing symptoms. For patients referred to the LAM Centre, rates of loss of lung function, disease related complications and deaths due to LAM were similar to that seen in sporadic LAM. Although the numbers of patients were relatively small, more deaths overall were seen in patients with TSC-LAM.

Our findings suggest that many patients with TSC are not being screened for LAM. Although screening had not been recommended for the first two years of the current study, it is clear many patients are only assessed for LAM when symptoms have developed. In addition, almost one third of patients were diagnosed with LAM after presentation with angiomyolipoma related symptoms, and whilst we don’t have data on angiomyolipoma screening for this group, this finding suggests that angiomyolipoma screening is also not performed in a significant number of patients with TSC. As mTOR inhibitors preserve lung function and probably increase survival, failure to actively screen for and treat LAM in TSC could allow the progression of irreversible, yet preventable lung damage and disability. In addition, bronchodilators improve airflow obstruction and early intervention in LAM can improve respiratory symptoms, alert patients to the risk of pneumothorax and also to avoid oestrogen containing treatments that can exacerbate disease [27]. Likewise, embolization or nephron sparing surgery to prevent angiomyolipoma bleeding and mTOR inhibitors to prevent tumour growth are effective and detecting angiomyolipoma is similarly important [28, 29]. In our current study and other series, angiomyolipoma are present in most patients with TSC-LAM [30, 31] and we would suggest that greater efforts are made to increase awareness of the need to screen for these manifestations of TSC and if angiomyolipoma is detected, then LAM should also be actively sought.

In our cohort, patients with TSC and sporadic LAM had broadly similar LAM symptoms and severity, although there was a significantly higher prevalence of angiomyolipoma and the need for angiomyolipoma intervention either with embolization, surgery or mTOR inhibitors in patients with TSC compared with sporadic LAM. Patients with TSC-LAM had slightly less advanced lung disease than sporadic LAM at presentation, being younger and less likely to present with dyspnoea although lung function wasn’t significantly different. Disease progression shown by rate of lung function loss, risk of death or need for lung transplant were similar in TSC and sporadic LAM. LAM was equally likely to be treated with an mTOR inhibitor with a similar benefit in preventing loss of FEV_1_ and DL_CO_ although an additional 17% of TSC-LAM patients were receiving an mTOR inhibitor primarily for angiomyolipoma rather than LAM. Whilst the incidence of pneumothorax was similar in both groups, surprisingly, in this cohort, patients with TSC-LAM were twice as likely to need surgery for pneumothorax. Although management guidelines for LAM are mostly based upon studies of sporadic LAM [25–27], the similar disease manifestations of TSC-LAM suggest that their use in TSC is appropriate; particularly recommendations for mTOR inhibitor therapy and early surgical treatment for pneumothorax [26, 27].

We noted our TSC-LAM cohort tended to be diagnosed with TSC later than average for all patients with TSC represented in the TOSCA study. They were also less likely to have epilepsy or neuropsychiatric complications. Whilst this may be referral bias, due to patients with severe intellectual disability being less likely to complain of respiratory symptoms or be referred to a pulmonary centre, it is also possible that TSC patients with milder neurological features may be more at risk of LAM and angiomyolipoma and consequently less likely to be diagnosed with TSC in childhood. Interestingly, no men with TSC were referred because of LAM. Although CT series report that lung cysts consistent with LAM occur in 10–30% of men this seldom leads to symptoms and for this reason, men with TSC are not screened for LAM and symptomatic disease appears uncommon [9, 32]. We have previously identified symptom clusters or sub-phenotypes in patients with LAM [30] and this may be also true in TSC but needs further study to determine the associations and predictors of the LAM/angiomyolipoma predominant patient with TSC. As the management of childhood TSC and life expectancy improve, LAM is becoming a larger part of TSC care and the morbidity of LAM makes it an important aspect of the TSC phenotype in adults [33]. Our findings suggest that respiratory physicians should be incorporated within the growing number of multidisciplinary TSC clinics and LAM screening should be actively pursued in this setting to address this challenge [34, 35].

Our study represents a national prospective audit of patients with TSC-LAM however has some limitations. Despite including a relatively large group of patients with TSC-LAM, this is a rare disease and numbers, particularly in some sub-group analyses, are small and subject to error. In addition, patients unable to travel to the UK LAM Centre could not be included which may limit representation of some subject groups, including those with more severe disability who may find it harder to travel to a specialist centre.

In conclusion, LAM is generally diagnosed in adult women with TSC after symptoms develop rather than by screening which may delay interventions to prevent loss of lung function. Complications of LAM including pneumothorax, lung function loss and death are significant in TSC-LAM and respiratory physicians should be more involved in TSC care. Improvements in implementing the recommended CT screening for LAM in TSC are required to ensure that appropriate advice is given and mTOR inhibitors are started as soon as there is evidence of progressive lung disease [20].

## Data Availability

Full audit data are presented in the manuscript.
